# 2,3,5,6-Tetra­fluoro-1,4-bis­(trimethyl­sil­yl)benzene

**DOI:** 10.1107/S1600536812010677

**Published:** 2012-03-17

**Authors:** Maik Finze, Guido J. Reiss, Hermann-Josef Frohn

**Affiliations:** a Institut für Anorganische Chemie, Julius-Maximilians-Universität Würzburg, Am Hubland, D-97074 Würzburg, Germany; b Institut für Anorganische Chemie und Strukturchemie, Lehrstuhl II: Material- und Strukturforschung, Heinrich-Heine-Universität Düsseldorf, Universitätsstrasse 1, D-40225 Düsseldorf, Germany; c Institut für Anorganische Chemie, Universität Duisburg-Essen, Lotharstrasse 1, D-47048 Duisburg, Germany

## Abstract

The asymmetric unit of the title compound, C_12_H_18_F_4_Si_2_, contains two independent mol­ecules, both lying on inversion centers. The C_arene_—Si distances are significantly longer than in the analogous non-fluorinated compound. The packing of the mol­ecules results in a herringbone motif in the *ac* plane.

## Related literature

For the synthesis and chemistry of 1,4-(Me_3_Si)_2_—C_6_F_4_, see: Fearon & Gilman (1967 ▶); Tamborski & Soloski (1969 ▶); Fields *et al.* (1970 ▶); Sartori & Frohn (1974 ▶); Bardin *et al.* (1991 ▶); Frohn *et al.* (1998 ▶); Kashiwabara & Tanaka (2006 ▶). For related structures see: Rehm *et al.* (1999 ▶); Sekiguchi *et al.* (2000 ▶); Haberecht *et al.* (2002 ▶, 2004 ▶); Krumm *et al.* (2005 ▶); Hanamoto *et al.* (2006 ▶).
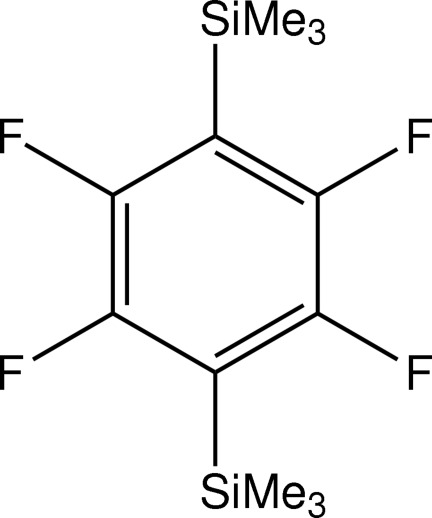


## Experimental

### 

#### Crystal data

C_12_H_18_F_4_Si_2_
*M* *_r_* = 294.44
Monoclinic, 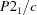

*a* = 19.8389 (4) Å
*b* = 6.35013 (10) Å
*c* = 12.3827 (2) Å
β = 107.407 (2)°
*V* = 1488.53 (5) Å^3^
*Z* = 4
Mo *K*α radiation
μ = 0.26 mm^−1^
*T* = 199 K
0.25 × 0.22 × 0.20 mm

#### Data collection

Oxford Xcalibur Eos diffractometer
8888 measured reflections
2626 independent reflections
2496 reflections with *I* > 2σ(*I*)
*R*_int_ = 0.015

#### Refinement

*R*[*F* ^2^ > 2σ(*F* ^2^)] = 0.027
*wR*(*F* ^2^) = 0.068
*S* = 1.09
2626 reflections
187 parameters
H-atom parameters constrained
Δρ_max_ = 0.39 e Å^−3^
Δρ_min_ = −0.22 e Å^−3^

### 

Data collection: *CrysAlis PRO* (Oxford Diffraction, 2009 ▶); cell refinement: *CrysAlis PRO*; data reduction: *CrysAlis PRO*; program(s) used to solve structure: *SHELXS97* (Sheldrick, 2008 ▶); program(s) used to refine structure: *SHELXL97* (Sheldrick, 2008 ▶); molecular graphics: *DIAMOND* (Brandenburg, 2011) ▶; software used to prepare material for publication: *publCIF* (Westrip, 2010 ▶).

## Supplementary Material

Crystal structure: contains datablock(s) I, global. DOI: 10.1107/S1600536812010677/mw2055sup1.cif

Structure factors: contains datablock(s) I. DOI: 10.1107/S1600536812010677/mw2055Isup2.hkl

Supplementary material file. DOI: 10.1107/S1600536812010677/mw2055Isup3.cml

Additional supplementary materials:  crystallographic information; 3D view; checkCIF report

## Figures and Tables

**Table 1:** Selected geometric parameters (Å, °)

Si1—C2	1.9101 (15)
Si2—C8	1.9077 (15)

**Fig. 1.**
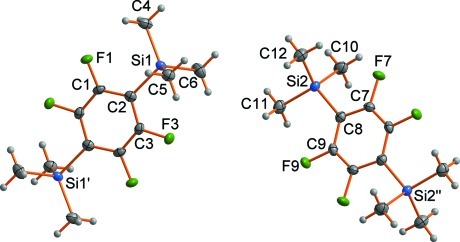
: The two crystallographically independent molecules of the title compound (H-atoms are drawn with arbitrary radii; ' = -x, 1 - y, -z; '' = 1 - x, 2 - y, 1 - z).

**Fig. 2.**
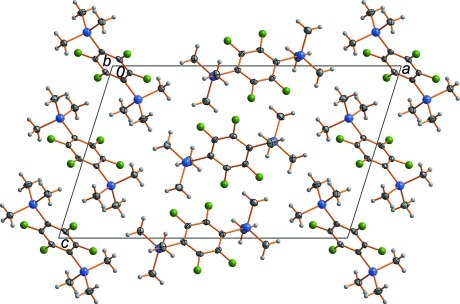
: Molecular packing of the title compound viewed parallel to the b axis, showing the herringbone type motif (ball and stick type model with arbitrary atom radii).

## supplementary crystallographic information

### Comment

The first synthesis of the title compound 1,4-bis(trimethylsilyl)tetrafluorobenzene, 1,4-(Me_3_Si)_2_—C_6_F_4_, was reported in 1967 starting from 1,2,4,5-tetrafluorobenzene, *n*-butyl lithium, and trimethylsilyl chloride (Fearon & Gilman 1967). Later, the compound was observed as a by-product in related reactions, improved methods for its selective synthesis were published and some reactions of 1,4-(Me_3_Si)_2_—C_6_F_4_ were described (Tamborski & Soloski, 1969; Fields *et al.* 1970; Sartori & Frohn, 1974; Bardin *et al.* 1991; Frohn *et al.* 1998; Kashiwabara & Tanaka, 2006). The 1,4-(Me_3_Si)_2_—C_6_F_4_ employed in the present study was prepared by a different route using poly(cadmium-2,3,5,6-tetrafluorobenzene), [1,4-Cd—C_6_F_4_]_n_, and trimethylsilyl chloride as starting materials.

The title compound 1,4-bis(trimethylsilyl)tetrafluorobenzene (Figure 1) crystallizes in the monoclinic space group *P*2_1_/*c* with two independent molecules each of which is located on a center of symmetry. Both crystallographically independent molecules display very similar geometric parameters. The C–C, C_methyl_–Si and C–F bond lengths are in the expected range. Both C_arene_–Si distances are slightly longer than those found for the non-fluorinated analogue 1,4-(Me_3_Si)_2_—C_6_H_4_ [*d*(C_arene_–Si) = 1.8817 (12) Å] (Haberecht *et al.* 2004) and other non-fluorinated Me_3_Si–C_arene_ compounds (Haberecht *et al.* 2004; Rehm *et al.* 1999). In contrast, for related 1-trimethylsilyl-2,3,5,6-tetrafluoro benzene fragments similar values were reported (Sekiguchi *et al.* 2000, Krumm *et al.* 2005). In the closely related compound 1,2,4-(*i*Pr_3_Si)_3_—C_6_F_3_ (Hanamoto *et al.* 2006) the *d*(C_arene_–Si) of the *i*Pr_3_Si groups in *ortho* positions [*d*(C–Si) = 1.937 (2), 1.934 (2)] are significantly longer than those in the title compound whereas the third C_arene_–Si distance [*d*(C–Si) = 1.914 (2) Å], which corresponds to the *i*Pr_3_Si group that has two F atoms in the *ortho* positions, is close to the values determined for 1,4-(Me_3_Si)_2_—C_6_F_4_.

The 1,4-bis(trimethylsilyl)tetrafluorobenzene molecules are arranged in the *ac* plane to form a herringbone structure (Figure 2). The trimethylsilyl groups are interlinked by van der Waals interactions with methyl groups of neighboring molecules.

### Experimental

The starting material poly(cadmium-2,3,5,6-tetrafluorobenzene), [1,4-Cd—C_6_F_4_]_n_, was synthesized by thermolysis of Cd(1,4-O_2_C—C_6_F_4_) at 270 °C under vacuum according to a literature procedure (Sartori & Frohn, 1974).

7.1 g (27.3 mmol) poly(cadmium-2,3,5,6-tetrafluorobenzene), [1,4-Cd—C_6_F_4_]_n_, was charged into a Duran-glass Carius tube inside a glove box. 6.55 g (60.3 mmol) freshly distilled (CH_3_)_3_SiCl was added under protection of dry nitrogen. The tube was sealed and shaken and heated inside an oven. The temperature was increased stepwise over 30 h to 222°C without visual change of the reaction components. Further heating from 230 to 250°C over 26 h was accompanied by a reduction of the liquid phase and a change of the color to light grey. The Carius tube was cooled stepwise to -78°C before opening under nitrogen protection. CAUTION: Handling of the sealed Carius tube should proceed behind a large protection screen with long-sleeve leather gloves. At 0°C 3.4 g (31.3 mmol) of (CH_3_)_3_SiCl were recovered by condensation under high-vacuum. The dark grey solid residue, which contained the co-product CdCl_2_, was extracted with boiling petrol ether (60–70°C fraction). After removing the solvent from the extract a slightly brownish oil remained which was sublimed under high vacuum. The colorless crystals were collected on a water cooled sublimation finger. Yield *ca* 80%; mp 46°C [31–32°C isomeric mixture (Fields *et al.*, 1970)]; NMR (20% CCl_4_ solution): ^1^H -0.39 p.p.m., ^19^F -124.2 p.p.m.; MS (EI, 73 eV) *M*^+.^ 294, the fragment ions 81 (CH_3_SiF_2_), 77 ((CH_3_)_2_SiF), and 73 ((CH_3_)_3_Si) possessed higher intensities than the parent ion; IR (neat): 2946 (*m*), 2889 (*m*), 1575 (w), 1487 (w), 1400 (st, b), 1343 (w), 1334 (w), 1286 (w), 1240 (st), 1214 (st), 1183 (*m*), 1035 (w), 921 (st), 830 (st, b), 748 (st), 684 (*m*), 613 (*m*), 566 (w), 432 (w).

### Refinement

Methyl H atoms were identified in a difference map, idealized and refined using rigid groups allowed to rotate about the Si—C bond (AFIX 137 option of the *SHELXL97* program). All *U*_iso_(H) values were refined unrestrictedly.

### Crystal data

**Table:**

C_12_H_18_F_4_Si_2_	*F*(000) = 616
*M**_r_* = 294.44	*D*_x_ = 1.314 Mg m^−^^3^
Monoclinic, *P*2_1_/*c*	Mo *K*α radiation, λ = 0.71073 Å
Hall symbol: -P 2ybc	Cell parameters from 8162 reflections
*a* = 19.8389 (4) Å	θ = 3.1–28.3°
*b* = 6.35013 (10) Å	µ = 0.26 mm^−^^1^
*c* = 12.3827 (2) Å	*T* = 199 K
β = 107.407 (2)°	Block, colourless
*V* = 1488.53 (5) Å^3^	0.25 × 0.22 × 0.20 mm
*Z* = 4	

### Data collection

**Table:**

Oxford Xcalibur Eos diffractometer	2496 reflections with *I* > 2σ(*I*)
Radiation source: fine-focus sealed tube	*R*_int_ = 0.015
Equatorial mounted graphite monochromator	θ_max_ = 25.0°, θ_min_ = 3.2°
Detector resolution: 16.2711 pixels mm^-1^	*h* = −22→23
ω–scan	*k* = −7→7
8888 measured reflections	*l* = −14→12
2626 independent reflections	

### Refinement

**Table:**

Refinement on *F*^2^	Primary atom site location: structure-invariant direct methods
Least-squares matrix: full	Secondary atom site location: difference Fourier map
*R*[*F*^2^ > 2σ(*F*^2^)] = 0.027	Hydrogen site location: inferred from neighbouring sites
*wR*(*F*^2^) = 0.068	H-atom parameters constrained
*S* = 1.09	*w* = 1/[σ^2^(*F*_o_^2^) + (0.0284*P*)^2^ + 1.0425*P*] where *P* = (*F*_o_^2^ + 2*F*_c_^2^)/3
2626 reflections	(Δ/σ)_max_ < 0.001
187 parameters	Δρ_max_ = 0.39 e Å^−^^3^
0 restraints	Δρ_min_ = −0.22 e Å^−^^3^

### Special details

**Table:**

Geometry. All esds (except the esd in the dihedral angle between two l.s. planes) are estimated using the full covariance matrix. The cell esds are taken into account individually in the estimation of esds in distances, angles and torsion angles; correlations between esds in cell parameters are only used when they are defined by crystal symmetry. An approximate (isotropic) treatment of cell esds is used for estimating esds involving l.s. planes.
Refinement. Refinement of *F*^2^ against ALL reflections. The weighted *R*-factor *wR* and goodness of fit *S* are based on *F*^2^, conventional *R*-factors *R* are based on *F*, with *F* set to zero for negative *F*^2^. The threshold expression of *F*^2^ > σ(*F*^2^) is used only for calculating *R*-factors(gt) *etc*. and is not relevant to the choice of reflections for refinement. *R*-factors based on *F*^2^ are statistically about twice as large as those based on *F*, and *R*- factors based on ALL data will be even larger.

### Fractional atomic coordinates and isotropic or equivalent isotropic displacement parameters (Å^2^)

**Table:**

	*x*	*y*	*z*	*U*_iso_*/*U*_eq_	
Si1	0.12486 (2)	0.23395 (7)	0.19607 (3)	0.01427 (11)	
C1	−0.01679 (8)	0.3116 (2)	0.03692 (12)	0.0145 (3)	
F1	−0.03838 (4)	0.12566 (13)	0.06873 (7)	0.0203 (2)	
C2	0.05291 (8)	0.3765 (2)	0.08187 (12)	0.0139 (3)	
C3	0.06680 (7)	0.5691 (2)	0.04012 (12)	0.0141 (3)	
F3	0.13382 (4)	0.64624 (14)	0.07568 (7)	0.0199 (2)	
C4	0.08544 (8)	0.0143 (3)	0.25580 (13)	0.0224 (3)	
H4A	0.0484	0.0679	0.2834	0.034 (5)*	
H4B	0.1212	−0.0499	0.3170	0.037 (5)*	
H4C	0.0663	−0.0886	0.1979	0.037 (5)*	
C5	0.19335 (8)	0.1412 (3)	0.13233 (13)	0.0190 (3)	
H5A	0.2119	0.2592	0.1019	0.033 (5)*	
H5B	0.1727	0.0427	0.0728	0.030 (5)*	
H5C	0.2309	0.0738	0.1894	0.037 (5)*	
C6	0.16406 (9)	0.4291 (3)	0.30949 (13)	0.0241 (4)	
H6A	0.1858	0.5406	0.2794	0.044 (6)*	
H6B	0.1990	0.3611	0.3703	0.045 (6)*	
H6C	0.1276	0.4864	0.3373	0.042 (6)*	
Si2	0.35988 (2)	0.79571 (7)	0.56124 (3)	0.01519 (11)	
C7	0.50606 (8)	0.8812 (2)	0.59251 (12)	0.0156 (3)	
F7	0.51552 (5)	0.76178 (15)	0.68697 (7)	0.0227 (2)	
C8	0.43772 (8)	0.9180 (2)	0.52347 (12)	0.0147 (3)	
C9	0.43451 (7)	1.0409 (2)	0.42942 (12)	0.0152 (3)	
F9	0.37103 (4)	1.08933 (14)	0.35422 (7)	0.0203 (2)	
C10	0.36292 (9)	0.8818 (3)	0.70638 (13)	0.0235 (4)	
H10A	0.3604	1.0326	0.7086	0.038 (6)*	
H10B	0.4063	0.8348	0.7594	0.032 (5)*	
H10C	0.3237	0.8222	0.7259	0.039 (6)*	
C11	0.27524 (8)	0.8855 (3)	0.46024 (13)	0.0214 (3)	
H11A	0.2727	0.8394	0.3853	0.031 (5)*	
H11B	0.2729	1.0364	0.4617	0.030 (5)*	
H11C	0.2365	0.8269	0.4816	0.034 (5)*	
C12	0.36891 (9)	0.5045 (3)	0.55206 (14)	0.0235 (4)	
H12A	0.3661	0.4662	0.4758	0.042 (6)*	
H12B	0.3316	0.4365	0.5734	0.049 (6)*	
H12C	0.4137	0.4610	0.6022	0.036 (5)*	

### Atomic displacement parameters (Å^2^)

**Table:**

	*U*^11^	*U*^22^	*U*^33^	*U*^12^	*U*^13^	*U*^23^
Si1	0.0148 (2)	0.0168 (2)	0.0121 (2)	0.00203 (16)	0.00533 (16)	0.00124 (16)
C1	0.0186 (7)	0.0128 (7)	0.0152 (7)	−0.0018 (6)	0.0099 (6)	0.0001 (6)
F1	0.0192 (5)	0.0163 (5)	0.0258 (5)	−0.0034 (4)	0.0073 (4)	0.0062 (4)
C2	0.0158 (7)	0.0160 (7)	0.0121 (7)	0.0017 (6)	0.0074 (6)	−0.0015 (6)
C3	0.0114 (7)	0.0177 (7)	0.0144 (7)	−0.0023 (6)	0.0057 (6)	−0.0030 (6)
F3	0.0124 (4)	0.0215 (5)	0.0247 (5)	−0.0043 (4)	0.0041 (4)	0.0018 (4)
C4	0.0210 (8)	0.0264 (9)	0.0213 (8)	0.0043 (7)	0.0089 (6)	0.0093 (7)
C5	0.0189 (8)	0.0216 (8)	0.0186 (7)	0.0019 (6)	0.0085 (6)	0.0008 (6)
C6	0.0262 (9)	0.0271 (9)	0.0162 (8)	0.0050 (7)	0.0019 (7)	−0.0033 (7)
Si2	0.0138 (2)	0.0159 (2)	0.0169 (2)	−0.00165 (16)	0.00616 (16)	−0.00042 (16)
C7	0.0187 (8)	0.0144 (7)	0.0135 (7)	0.0006 (6)	0.0045 (6)	0.0025 (6)
F7	0.0190 (5)	0.0288 (5)	0.0191 (5)	−0.0001 (4)	0.0039 (4)	0.0117 (4)
C8	0.0156 (7)	0.0134 (7)	0.0156 (7)	−0.0005 (6)	0.0055 (6)	−0.0020 (6)
C9	0.0122 (7)	0.0159 (7)	0.0153 (7)	0.0020 (6)	0.0006 (6)	−0.0009 (6)
F9	0.0124 (4)	0.0260 (5)	0.0192 (4)	0.0008 (4)	0.0000 (3)	0.0064 (4)
C10	0.0260 (9)	0.0264 (9)	0.0209 (8)	−0.0044 (7)	0.0116 (7)	−0.0012 (7)
C11	0.0157 (8)	0.0254 (9)	0.0240 (8)	−0.0011 (6)	0.0075 (6)	−0.0006 (7)
C12	0.0237 (8)	0.0185 (8)	0.0299 (9)	−0.0019 (7)	0.0105 (7)	0.0003 (7)

### Geometric parameters (Å, º)

**Table:**

Si1—C4	1.8574 (16)	Si2—C11	1.8579 (16)
Si1—C5	1.8595 (15)	Si2—C10	1.8620 (16)
Si1—C6	1.8602 (16)	Si2—C12	1.8644 (17)
Si1—C2	1.9101 (15)	Si2—C8	1.9077 (15)
C1—F1	1.3539 (17)	C7—F7	1.3591 (17)
C1—C3^i^	1.379 (2)	C7—C9^ii^	1.378 (2)
C1—C2	1.390 (2)	C7—C8	1.389 (2)
C2—C3	1.387 (2)	C8—C9	1.387 (2)
C3—F3	1.3605 (16)	C9—F9	1.3587 (16)
C3—C1^i^	1.379 (2)	C9—C7^ii^	1.378 (2)
C4—H4A	0.9600	C10—H10A	0.9600
C4—H4B	0.9600	C10—H10B	0.9600
C4—H4C	0.9600	C10—H10C	0.9600
C5—H5A	0.9600	C11—H11A	0.9600
C5—H5B	0.9600	C11—H11B	0.9600
C5—H5C	0.9600	C11—H11C	0.9600
C6—H6A	0.9600	C12—H12A	0.9600
C6—H6B	0.9600	C12—H12B	0.9600
C6—H6C	0.9600	C12—H12C	0.9600
			
C4—Si1—C5	112.29 (7)	C11—Si2—C10	108.76 (7)
C4—Si1—C6	109.29 (8)	C11—Si2—C12	110.31 (8)
C5—Si1—C6	109.76 (7)	C10—Si2—C12	111.96 (8)
C4—Si1—C2	109.89 (7)	C11—Si2—C8	110.18 (7)
C5—Si1—C2	108.36 (7)	C10—Si2—C8	108.80 (7)
C6—Si1—C2	107.11 (7)	C12—Si2—C8	106.81 (7)
F1—C1—C3^i^	117.10 (13)	F7—C7—C9^ii^	117.62 (13)
F1—C1—C2	120.37 (13)	F7—C7—C8	118.79 (13)
C3^i^—C1—C2	122.53 (14)	C9^ii^—C7—C8	123.58 (14)
C3—C2—C1	113.39 (13)	C9—C8—C7	113.74 (13)
C3—C2—Si1	120.39 (11)	C9—C8—Si2	126.70 (11)
C1—C2—Si1	126.13 (11)	C7—C8—Si2	119.55 (11)
F3—C3—C1^i^	117.22 (13)	F9—C9—C7^ii^	117.13 (13)
F3—C3—C2	118.72 (13)	F9—C9—C8	120.19 (13)
C1^i^—C3—C2	124.06 (13)	C7^ii^—C9—C8	122.68 (13)
Si1—C4—H4A	109.5	Si2—C10—H10A	109.5
Si1—C4—H4B	109.5	Si2—C10—H10B	109.5
H4A—C4—H4B	109.5	H10A—C10—H10B	109.5
Si1—C4—H4C	109.5	Si2—C10—H10C	109.5
H4A—C4—H4C	109.5	H10A—C10—H10C	109.5
H4B—C4—H4C	109.5	H10B—C10—H10C	109.5
Si1—C5—H5A	109.5	Si2—C11—H11A	109.5
Si1—C5—H5B	109.5	Si2—C11—H11B	109.5
H5A—C5—H5B	109.5	H11A—C11—H11B	109.5
Si1—C5—H5C	109.5	Si2—C11—H11C	109.5
H5A—C5—H5C	109.5	H11A—C11—H11C	109.5
H5B—C5—H5C	109.5	H11B—C11—H11C	109.5
Si1—C6—H6A	109.5	Si2—C12—H12A	109.5
Si1—C6—H6B	109.5	Si2—C12—H12B	109.5
H6A—C6—H6B	109.5	H12A—C12—H12B	109.5
Si1—C6—H6C	109.5	Si2—C12—H12C	109.5
H6A—C6—H6C	109.5	H12A—C12—H12C	109.5
H6B—C6—H6C	109.5	H12B—C12—H12C	109.5
			
F1—C1—C2—C3	179.45 (12)	F7—C7—C8—C9	−179.74 (13)
C3^i^—C1—C2—C3	−1.0 (2)	C9^ii^—C7—C8—C9	0.1 (2)
F1—C1—C2—Si1	−3.9 (2)	F7—C7—C8—Si2	−1.36 (19)
C3^i^—C1—C2—Si1	175.56 (11)	C9^ii^—C7—C8—Si2	178.52 (12)
C4—Si1—C2—C3	167.71 (11)	C11—Si2—C8—C9	−5.61 (16)
C5—Si1—C2—C3	−69.26 (13)	C10—Si2—C8—C9	−124.75 (14)
C6—Si1—C2—C3	49.09 (13)	C12—Si2—C8—C9	114.22 (14)
C4—Si1—C2—C1	−8.68 (15)	C11—Si2—C8—C7	176.25 (12)
C5—Si1—C2—C1	114.36 (13)	C10—Si2—C8—C7	57.10 (14)
C6—Si1—C2—C1	−127.29 (13)	C12—Si2—C8—C7	−63.93 (13)
C1—C2—C3—F3	−178.22 (12)	C7—C8—C9—F9	179.85 (13)
Si1—C2—C3—F3	4.96 (18)	Si2—C8—C9—F9	1.6 (2)
C1—C2—C3—C1^i^	1.1 (2)	C7—C8—C9—C7^ii^	−0.1 (2)
Si1—C2—C3—C1^i^	−175.76 (11)	Si2—C8—C9—C7^ii^	−178.38 (11)

Symmetry codes: (i) −*x*, −*y*+1, −*z*; (ii) −*x*+1, −*y*+2, −*z*+1.
